# Racism as a public health issue in environmental health disparities and environmental justice: working toward solutions

**DOI:** 10.1186/s12940-024-01052-8

**Published:** 2024-01-22

**Authors:** Sharon Beard, Kenda Freeman, Maria L. Velasco, Windy Boyd, Toccara Chamberlain, Alfonso Latoni, Denise Lasko, Ruth M. Lunn, Liam O’Fallon, Joan Packenham, Melissa M. Smarr, Robin Arnette, Crystal Cavalier-Keck, Jason Keck, Naeema Muhammad, Omega Wilson, Brenda Wilson, Ayo Wilson, Darlene Dixon

**Affiliations:** 1 Division of Extramural Research and Training, National Institute of Environmental Health Sciences, Durham, North Carolina USA; 2 MDB Inc, Durham, North Carolina USA; 3 Office of the Director, National Institute of Environmental Health Sciences, Durham, North Carolina USA; 4 Division of Translational Toxicology, National Institute of Environmental Health Sciences, Mail Drop B3-06 Rall Bldg. 101, Rm. B341, P.O. Box 12233, Durham, North Carolina 27709 USA; 5 Division of Intramural Research, National Institute of Environmental Health Sciences, Durham, North Carolina USA; 6 Office of Communications and Public Liaison, National Institute on Aging, Bethesda, Maryland USA; 7 7 Directions of Service, Mebane, North Carolina USA; 8 North Carolina Environmental Justice Network, Raleigh, North Carolina USA; 9 West End Revitalization Association, Mebane, North Carolina USA

**Keywords:** Environmental justice, Environmental health disparities, Systemic racism, Toxic exposures, Solutions

## Abstract

**Background:**

Environmental health research in the US has shown that racial and ethnic minorities and members of low-socioeconomic groups, are disproportionately burdened by harmful environmental exposures, in their homes, workplace, and neighborhood environments that impact their overall health and well-being. Systemic racism is a fundamental cause of these disproportionate exposures and associated health effects. To invigorate and inform current efforts on environmental justice and to raise awareness of environmental racism, the National Institute of Environmental Health Sciences (NIEHS) hosted a workshop where community leaders, academic researchers, and NIEHS staff shared perspectives and discussed ways to inform future work to address health disparities.

**Objectives:**

To share best practices learned and experienced in partnerships between academic researchers and communities that are addressing environmental racism across the US; and to outline critical needs and future actions for NIEHS, other federal agencies, and anyone who is interested in conducting or funding research that addresses environmental racism and advances health equity for all communities.

**Discussion:**

Through this workshop with community leaders and researchers funded by NIEHS, we learned that partnerships between academics and communities hold great promise for addressing environmental racism; however, there are still profound obstacles. To overcome these barriers, translation of research into plain language and health-protective interventions is needed. Structural changes are also needed in current funding mechanisms and training programs across federal agencies. We also learned the importance of leveraging advances in technology to develop creative solutions that can protect public health.

## Introduction

### NIEHS efforts focused on environmental health disparities

Many people across the U.S. are exposed to toxic waste and other hazardous substances, but some communities bear the burden of these exposures, and associated diseases, more than others. Individuals have different exposures, susceptibilities, and resistance to disease, based on unique social, biological, and environmental forces over their life course [1–3].

America is segregated, and so is pollution. Race has been the most potent factor in deciding where toxic facilities are located. Racial and ethnic minorities and low-income groups often live in neighborhoods near hazardous waste and are disproportionately burdened with environmental pollution [4, 5]. Environmental racism includes the use of racist and discriminatory practices in determining which communities receive either health-protective or health-harming policies, practices, and infrastructure [6, 7]. The social system that creates discrimination and inequalities is also responsible for patterns of disease [1, 3].

For decades, the 17 principles of environmental justice [8] have served as guidance for federal agencies and community-based organizations to ensure that all people and communities are entitled to equal protection of the environment, education, energy, health, and employment regardless of race, color, national origin, or income. Federal agencies like the National Institutes of Health (NIH) have adopted these principles as a basis for research, training, and educational initiatives. For example, the National Institute of Environmental Health Sciences (NIEHS) has been focused on environmental justice since the early 1990s. Former NIEHS Director Kenneth Olden, Ph.D., contributed to the establishment of community-based and community-participatory models to study environmental hazards and their health impacts on communities of color facing disproportionate environmental exposures [9, 10]. Additionally, Dr. Olden initiated NIEHS Community Forums that promoted opportunities to hear from and listen to residents of North Carolina, where NIEHS is located, as a way of understanding what their needs were and how the Institute could best respond to those needs [11]. Several NIEHS staff have followed the lead of Olden, such as former NIEHS Director Linda Birnbaum, Ph.D., and others interested in environmental health disparities. Current NIEHS director, Rick Woychik, Ph.D. has expressed his commitment to prioritize the Institute’s focus on environmental justice, health disparities, and community engagement.

In 2020, NIEHS established the Environmental Health Disparities and Environmental Justice (EHD and EJ) Faculty to advance environmental justice research that will promote environmental health equity [12]. The working group, which brings together staff members from across NIEHS, facilitates in-depth discussions about tangible responses to environmental justice issues. These include promoting new approaches to study the impact of environmental racism on health, increasing interaction and collaboration across NIEHS and with communities disproportionately exposed to contamination, and elevating environmental justice work across NIEHS research and training activities.

### NIEHS workshop addresses issues of racism and environmental health disparities

As a first step to build awareness of environmental justice issues in the context of systemic racism, NIEHS hosted a virtual workshop on December 10, 2021, “Addressing Racism as a Public Health Issue Through the Lens of Environmental Health Disparities and Environmental Justice.” The workshop was organized by a committee made up of the NIEHS EHD and EJ Faculty, as well as community partners West End Revitalization Association (WERA), North Carolina Environmental Justice Network (NCEJN), and 7 Directions of Service. These community partners have been environmental justice leaders in North Carolina for decades and provided valuable planning input to ensure the workshop reflected the views and needs of the communities they serve. In addition to the community leaders involved in planning the workshop, speakers and panelists included NIEHS staff, environmental justice leaders in academia, NIEHS-funded academic researchers, and community-based organizations that collaborate with NIEHS-funded researchers. Through keynote presentations and panel discussions, speakers shared research and community-based perspectives and discussed ways to inform research and outreach activities that address environmental health inequities.

The overarching goals of the workshop were to: raise awareness of the problem of structural racism in America and its contributing role to environmental health disparities; guide plans and recommendations to integrate into NIEHS’ portfolio of research and outreach activities; implement these plans and recommendations to guide efforts to address the challenges associated with environmental injustices; and engage local, regional, and national community leaders and advocates to discuss best practices for community engagement.

The purpose of this commentary is to summarize the key takeaways and action plans identified during the workshop and provide recommendations to inform future plans for NIEHS and other federal agencies to address environmental injustices and health disparities. This manuscript will highlight the disproportionate environmental burdens that many communities face locally in North Carolina, regionally, and across the U.S. and will describe collaborations between communities and academic researchers that address these issues and promote health equity. The commentary will also highlight interactions between communities, academics, and federal agencies, providing a model to address systemic racism in environmental health research.

## Key environmental justice issues in communities

Workshop speakers shared their perspectives regarding environmental justice issues prevalent within their communities with the goal of inspiring new research and actions that address their needs and promote health equity. Community partners discussed how rural and urban minority communities face gentrification — social, cultural, political, and economic change to bring capital, people, and industries into communities that were previously segregated, redlined communities or in the case of tribes, communities protected by their sovereign rights. These communities actively fight against pipelines, mining projects, and highways that may impact their water and other environmental resources, as well as their ancestral traditions, their health, and their wellbeing.

In North Carolina, concentrated animal feeding operations (CAFOs), located in predominately Black communities and low-wealth areas [13], are associated with a myriad of environmental issues, including water and air pollution. These exposures have been associated with detrimental respiratory health effects, including asthma and lung function decline [14] and have been shown to impact the physical and mental health of nearby communities [15, 16]. Also, weather conditions such as hurricanes and other natural disasters add another layer of complexity to environmental exposures from CAFOs due to unintended distribution or re-distribution of waste. A community partner explained that many rural ethnic minority communities face compounding exposures to contaminants from CAFOs and natural disasters, which can have long-term health effects and lead to diseases that develop later in life, such as kidney disease [16].

In urban areas, low-wage, racial and ethnic minority communities are exposed to pollution from nearby railroads, highways, industries, municipal incinerators and other toxic facilities or waste sites, that harms their health and wellbeing. Some of these communities also lack adequate infrastructure, including aging infrastructure that threatens sewage and safe drinking water [17].

According to community partners, Native American and Indigenous communities are some of the most marginalized people in the U.S.; are often invisible and left out; and are often overlooked in data, communications, and decision-making. Native Americans are more vulnerable to the health effects of environmental pollution, such as asthma [18], due to their reliance on nature and the history of harmful mining practices on their land. For example, the rate of asthma in the Native American and tribal populations is almost double the national average [18].

Latino and immigrant populations experience another layer of complex social factors, such as employment insecurity and low wages, which increase their susceptibility for environmental exposures and health disparities. Immigrant workers, especially low-wage Latino laborers like household cleaning workers, are at higher risk for a range of work-related health issues, including toxic exposures, injuries, and negative mental health outcomes [19]. Social factors, including structural racism, language-related communication barriers with clients, negative client support, and food, housing, and financial insecurity exacerbate the effects of these environmental exposures [20, 21].

According to community partners, thousands of public schools in urban, small-city, rural, and Indigenous areas have an increased risk of experiencing environmental injustices. Chronic contamination in schools has been linked to respiratory and neurodevelopmental issues [22, 23]. Higher percentages of students of color are absent from school because of asthma, which can lead to lower academic performance. Extreme weather events also damage schools in coastal areas. Workshop speakers shared that schools in these and other rural areas often have compromised structures, such as leaky roofs; mold; mildew; and contaminants, such as lead. These pollutants create long-standing issues in schools, robbing children of a healthy atmosphere to develop and learn.

## Building partnerships in disproportionately impacted communities

According to the workshop speakers, successful environmental health research must follow anti-racism principles and promote anti-racist practices within partnerships and relationships. Based on insights shared by the workshop speakers, successful and effective partnerships are those that apply and demonstrate community-based participatory research (CBPR) in action; work to challenge systems that produce racial inequities in environmental exposures or health outcomes; ensure translation of research into tangible benefits for affected communities; and share power equitably between researchers and communities to address health inequities. NIEHS recognizes these principles as a foundation for future research activities, funding opportunities, guidelines, and policies.

CBPR incorporates input from the people who will be impacted by the research and involves people or groups as equal partners. The strategy builds upon the strengths and expertise of each partner to inform the research from conception to dissemination [24]. CBPR models can address environmental injustices and inequities, but there is no one-size-fits-all approach. Workshop speakers stated that an effective partnership must share power equitably and leverage a community’s influence to address underlying health inequities, including those that are linked to disproportionate environmental exposures. Effective CBPR promotes open and frequent communication between all partners, benefits all partners equally, and holds all partners accountable to fulfill their roles.

Speakers shared their experiences and ongoing work to conduct community-based participatory research that addresses environmental health disparities in diverse communities. The examples outlined below showcase CBPR in action and draw from key insights shared by academic researchers and their respective community partners during presentations. These examples also outline the challenges, successes, and approaches for creating equitable and successful partnerships between community-based organizations, community leaders and residents, and academic institutions.

### Partnerships with latino communities

Funded by the NIEHS Research to Action program, Make the Road New York (MRNY), a community-based organization in New York City, and academic researchers at Queens College, the Icahn School of Medicine at Mount Sinai, and the University of Massachusetts Lowell, use a collaborative participatory approach to protect the health of Latino household cleaning workers through the Safe and Just Cleaners project [25].

Community input is the foundation of the Safe and Just Cleaners project [20]. Together, the team of academic researchers along with MRNY staff prioritize gathering community input in the planning stages before conducting research or developing public health campaigns. This ensures that research and outreach activities address the community’s needs. For example, the team conducted focus groups, interviews, and surveys to learn about community challenges and needs. They learned about the physical and mental health challenges, as well as social stressors the cleaners were facing, including lack of health coverage, verbal abuse, job insecurity, and exposure to chemicals and irritants with acute health effects. They also learned about effective methods of communication, like who the community trusts to obtain accurate information.

While academic researchers offer expertise in identifying and measuring exposures, MRNY leads most of the community engagement efforts for the Safe and Just Cleaners project. MRNY builds the power of Latino and working-class communities through organizing, policy innovation, transformative education, and survival services. In partnership with project participants, MRNY has promoted action at different levels, including educational activities and insisting on helpful policies. For example, Las Super Cleaners Group, which was created by project participants, meets monthly to provide a space for household cleaning workers to connect and learn about environmental toxins. Organizing among participants has led to economic support for immigrants and undocumented workers during the COVID-19 pandemic [26].

Building successful relationships between communities, organizations, and academic researchers requires time and commitment. The partnership between MRNY, academic researchers, and Latino household cleaning workers was initiated in 2018. The partners continue to meet on a regular basis to share information about environmental toxins, safety at the workplace, workers’ rights, and organizing tools.

### Partnerships with urban communities

Community Action to Promote Healthy Environments (CAPHE), a community-based participatory research project, is working to improve air quality and health in Detroit. The project involves collaboration between community-based organizations, the healthcare community, environmental organizations, and academic researchers at the University of Michigan as equal partners and co-investigators. CAPHE is funded by the NIEHS Research to Action program [27].

Together, the CAPHE team developed a public health action plan that includes recommendations to reduce air pollution and adverse health effects [28]. To develop the action plan, the researchers mapped air pollutant levels, sources, and distribution, then quantified health impacts and inequities in the Detroit metropolitan area (DMA), which is among the most racially segregated regions in the nation and is home to mainly Black and Latino communities with low to moderate incomes [29]. Mapping conducted by the CAPHE team showed that although the areas outside the DMA consume more energy, the DMA has a higher health burden due to air pollution [30]. The researchers worked with community partners to examine strategies implemented in other cities that face similar air pollution issues, to determine recommendations that could be applied in the public health action plan for Detroit. Then, the collaborators held public community meetings to share their findings and listen to resident’s health concerns to refine their recommendations.

The plan details 25 actionable strategies based on science and community priorities, such as incorporating vegetative buffers, installing air filters, and improving infrastructure to allow for alternative transit forms like biking. This research informed new policies by the City of Detroit that require companies to conduct a health impact assessment before starting industrial activities.

Workshop speakers stated that this collaboration taught them the importance of ensuring that community leaders are co-investigators, not just collaborators, in research projects, through sharing responsibilities, leadership, and recognition. They also shared that self-reflexivity and evaluation throughout the process are essential for ensuring a project’s effectiveness. In fact, the team now conducts health impact assessments to understand the health impacts of proposed policies or projects before they are implemented.

Another urban partnership highlighted in the workshop featured the community-based organization WE ACT for Environmental Justice and researchers at Columbia University, who have long studied the impacts of environmental exposures, such as air pollution and bisphenol A, on human health. Based in New York City, WE ACT for Environmental Justice is a prominent leader in community organizing and advocacy on environmental justice issues.

Workshop speakers honored the late Cecil Corbin-Mark, who served with WE ACT for over 25 years and helped develop and pass numerous health-protective bills in New York. Together with Columbia University researchers, WE ACT has used research findings to inform policies, such as classifying waste from oil and gas as hazardous waste, resulting in the introduction of hybrid buses in New York City. The team also helped pass the strongest lead-poisoning law in the city, as well as a climate legislation to commit the state of New York to net-zero emissions by 2050 and require at least 35% of state energy and climate spending to go to pollution-burdened communities.

### Partnerships with native American tribes

There are over 500 abandoned uranium mines on Navajo Nation lands, exposing tribes to numerous environmental hazards, including heavy metals and particulate matter [31].

Researchers at the University of New Mexico partnered with the Navajo Nation Blue Gap-Tachee Chapter to learn about their exposure risks and help address their health concerns. This work is funded by the NIEHS Superfund Research Program and the Specialized Centers of Excellence on Environmental Health Disparities Research Program; the latter program is jointly supported by NIEHS, the National Institute on Minority Health and Health Disparities, and the Eunice Kennedy Shriver National Institute of Child Health and Human Development.

The partnership between researchers and the Navajo Nation helped build relationships between the tribe and federal agencies and facilitated development of a plan to protect tribal health. For example, the partners conducted environmental studies that led to the Navajo Nation Department of Justice and U.S. Environmental Protection Agency (EPA) Office to list Blue Gap-Tachee land as a priority cleanup site in 2014 [32, 33]. University of New Mexico researchers also hold regular discussions and scientific meetings with Navajo Nation and EPA. These discussions are helpful for conducting health and risk assessments to ensure that research leads to actions that benefit the tribe.

Other work by the partners found barriers in communication between scientists and tribal members, such as a lack of terms in the Navajo language to identify certain internal organs and chemicals. To address this need, the team frequently translates reports and educational materials to Navajo. Through community meetings they learned that there was some exposure and health related terminology that could not be translated to Navajo, so they worked with Navajo leaders to create the Navajo Health Dictionary, which includes new words to translate research terminology. They also collaborate with a local Navajo artist to create art that uses tribal symbolism to explain complex research concepts, such as how chemicals can damage cells and DNA [34].

As stated by the workshop speakers, partnerships between academics and Native American communities should be built on mutual trust and interests. Over the years, many University of New Mexico researchers have belonged to Native American tribes and their scientific talent, cultural awareness, and ability to build trusted partnerships have been vital to the success of this project [35].

### Partnerships with indigenous and Alaska tribes

The Alaska Community Action on Toxics (ACAT), a community-based organization, and academic researchers lead a collaborative project with the Indigenous Yupik people of St. Lawrence Island, known by the traditional name Sivuqaq, in Arctic Alaska. This project, funded by the NIEHS Research to Action program, is a collaboration between ACAT, the University of Arizona, Northern Arizona University, Middlebury College, the University of Albany, and Emory University [36]. Sivuqaq is home to two abandoned Cold War-era military bases, and is contaminated with PCBs, fuel, solvents, pesticides, lead, mercury, asbestos, and more. Emerging contaminants, such as per- and polyfluoroalkyl substances, have also been detected on the island [37].

In response to community advice, ACAT relies on the knowledge of tribal elders when planning and conducting research. ACAT also works with communities to collect environmental samples, conducts community health surveys, and maintains a cancer registry.

ACAT is investigating exposure to contaminants in the area and health effects. The organization works closely with Alaska Native tribes, healthcare professionals, students, and other residents to conduct community-based participatory research [38]. Tribal members, and other collaborators, are not only involved in data collection and analysis, but also in capacity-building events, such as workshops, webinars, and community forums to learn about best practices for conducting research, protecting and improving health, and collecting clinical health information.

Workshop speakers shared that when scientists are working with Indigenous communities, they are sometimes perceived as “researching on” instead of “researching with” the communities. Therefore, it is important for researchers to come with an open mind, ready to receive input from community members on their needs and how the work should be conducted.

## Recommendations for the future

Partnerships between academic researchers and community-based organizations hold great promise for improving public health for all communities, but particularly for those experiencing environmental racism and health disparities. However, there are profound obstacles to promoting environmental justice and conducting collaborative research that need to be addressed.

During moderated panel discussions, speakers identified common challenges and strategies for conducting environmental health research that can reduce health disparities for all communities. This information, summarized in Table 1, will provide a foundation for NIEHS and other federal agencies to promote environmental health research that addresses systemic racism and to ensure that anti-racist partnerships exist between academic researchers, federal agencies, and community-based organizations to support environmental justice in communities.

**Table 1:** Challenges and recommendations to addressing environmental racism in environmental health research, as discussed by workshop participants

Category	Challenge	Recommendation
Structural changes to funding mechanisms	The community’s needs are not considered until after a research project has been designed and often funded. This results in missed opportunities for gathering data that can be used to create tangible, positive change for affected communities.	Funding mechanisms should require that communities should be part of the discussion early in the planning phase (i.e., during development) of the research project, and their input should be integrated over the course of conducting the project.
Structural changes to funding mechanisms	Community partners are sometimes not adequately compensated for their contributions to projects.	Federal agencies should ensure that there is equitable distribution of funds between academia and community-based organizations in the application review process. In addition, funding should also go to diverse institutions and organizations that represent affected communities, such as historically black colleges and universities.
Structural changes to funding mechanisms	Representatives from the communities where research is being conducted do not have a say in the process of deciding which projects will receive funding.	Community-based organizations, environmental justice advocates, and investigators who are experienced in community-based participatory research should be part of review committees.
Communication, engagement, and research translation	Research results are often communicated in academic publications for scientific audiences.	Reports should be provided back to communities in plain language, allowing the people who have a stake in and who benefit from the research to understand the findings.
Communication, engagement, and research translation	Translating research into health-protective interventions can be a long, challenging process.	Results should be provided in a way that is accessible to multiple groups, not just the scientific community, and easy to translate into action steps health-protective interventions, including but not limited to policies, organizing, and educational activities.
Training	Traditional academic education often does not emphasize the value of using research to answer the community’s questions.	Early-stage investigators and students should receive training opportunities working with community-based organizations.
New technologies and data	The long-term health impacts of environmental racism can be difficult to track.	Community-based organizations, federal agencies, and academics should work together to document environmental justice trends and protect public health.

### Communication, engagement, and research translation

The speakers believe academic researchers have a duty to use science in the name of justice and equity and to work with community partners to understand and address their issues. Engaging with communities is key to ensuring that scientific research can be translated into actions that protect the health of communities disproportionately affected by environmental pollution. However, conversations in this workshop suggested that to ensure successful relationships with communities, researchers must develop partnerships with local and regional environmental justice leaders. Trusted environmental justice leaders can help academic researchers understand the specific concerns and needs of communities and develop effective strategies to address those needs.

Community partners noted researchers often write for academic audiences, but scientific research is most useful when translated into plain language. Doing so allows the people who have a stake in and who benefit from the research to understand the findings. For example, researchers should incorporate traditional ecological knowledge and visuals when sharing scientific results with Native American and Indigenous communities. Researchers should also consider other avenues to communicate their research in a timely manner, including public television, social media, and frequent in-person or virtual forums with the community.

Academic research can also inform new policies to protect the health and wellbeing of communities. Research reports should be translated in a way that will inform future policies and lead to successful policy-making discussions. Researchers can also participate in strengthening community-based organizations and their environmental justice efforts by providing research support, technical assistance, education, and financial backing.

### Training

Training the next generation of environmental health scientists is an important component of NIEHS, especially of its Division of Extramural Research and Training. Community partners expressed that researchers should understand the community’s need for answers, so trainees must learn how to use research to answer their questions and to support the community moving forward.

Looking to the future, academic researchers should include training opportunities with community-based organizations and community-based participatory research projects and should stress two-way communication with the community. Researchers should also emphasize the importance of bringing the community into discussions early in the planning process and creating a safe space for the community to share needs throughout the research project.

Health care professionals are important partners in the advancement of environmental health, as they play a key role in providing patients and communities with valuable information. Training and communication strategies should also be shared with health care professionals, so they can learn how to build environmental health literacy about exposures within their constituencies. In the case of Native American communities, health care professionals must be trained on how to integrate western health care systems into traditional ecological knowledge [39].

### Leveraging new technologies and data

Environmental justice problems, and their solutions, are complex and cross the boundaries of scientific disciplines. Integrating datasets from community scientists, academic institutions, and state and federal agencies can help address these needs and help move research forward and protect human health. These data can also be used to inform decisions that affect public health. While community science data is important for environmental health research and decision-making, it is often underused due to inconsistent collection methods and interoperability concerns. Advances in data science and data sharing can help address these needs and reveal new information that could have not been discovered before [40].

While sharing data can help provide new insights, it is important to recognize that not all data can, or needs to, be shared. As stated by community partners, academic researchers need to ensure data is correctly masked for all populations. Just like data sovereignty protects tribal data, this approach needs to be used to protect and respect the data privacy of other communities, such as immigrant and displaced populations.

Monitoring tools can help track and document long-term health impacts for people living in communities impacted by climate-related disasters. For example, mapping tools can show trends across environmental contaminants, sociodemographic factors, environmental justice indicators, and health outcomes, uncovering patterns that community-based organizations can show to legislators and other agencies to advocate for environmental justice. Mapping tools, and the insights gained through them, may also be used to retrospectively understand what actions have or have not worked in ensuring public health and well-being for all.

Going forward, community-based organizations, federal agencies, and academic institutions should work with each new generation of researchers who have expertise in novel technologies to create tools to protect public health, and to collaborate with other agencies to develop creative solutions that cross the boundaries of multiple scientific disciplines.

### Structural changes to NIEHS funding mechanisms

Workshop speakers maintained that there are few structural mechanisms to support non-science activities of community-based organizations, such as educational campaigns. During moderated discussions it was expressed that traditional funding models do not directly support community-based organizations as it is usually up to the discretion of the academic institution awarded the grant to distribute funds with collaborators. The community leaders and academic researchers also shared that a five-year research project is not long enough to develop and implement health-protective actions, or to ensure these actions are sustainable. To address these hurdles, speakers called for funding mechanisms that expand community-based, participatory research programs and support the time and resources necessary to implement long-lasting actions in communities. NIEHS and federal agencies can help overcome these challenges by encouraging community-based organizations to pursue other types of grants and funding opportunities or by promoting partnerships with other agencies and organizations. Community partners also expressed a need for federal agencies to ensure equitable distribution of funds between academia and community-based organizations. It was suggested that this action should be a requirement, explicitly stated in federal requests for applications, and examined in the application review process, before making awards.

Federal agencies must also consider what funding or resources are needed to support the basic needs of the community, especially in the face of emerging challenges. For example, in response to the COVID-19 pandemic, the NIEHS Division of Extramural Research and Training released several supplemental funding opportunities through the Superfund Research Program and the Worker Training Program. NIEHS also signed onto the NIH Rapid Acceleration of Diagnostics (RADx) initiative to ensure that all communities have access to COVID-19 testing and surveillance [41].

Funding should also go to diverse institutions, such as Historically Black Colleges and Universities (HBCUs) and toward programs that ensure diversity and equity in faculty, staff, and students at other universities. An example of a strategy to ensure diversity, equity, and inclusion is the NIH UNITE Initiative, which NIEHS has committed to implement [42]. The initiative aims to identify strategies to end racism in a way that will be transformative for the biomedical and environmental health sciences workforce. As a part of this initiative, NIEHS aims to ensure transparency and communication with community partners, fund new research on health disparities, improve its own culture for equity and inclusion, and change the structure of academic research to promote workforce diversity. Another initiative, the NIEHS Scholars Connect Program, invites applications from HBCUs and other academic institutions to provide hands-on, mentored research experiences for undergraduate students, to encourage them to pursue careers in scientific investigation [43].

Community partners also expressed a need for a shift in decision-making in existing and new research programs. Federal agencies and universities need to make sure that community-based organizations, environmental justice advocates, and investigators who are experienced in community-based participatory research are part of review committees, to ensure new research projects address environmental health disparities.

Community-based organizations are not always the beneficiaries of university-led research, speakers shared, because the university usually manages each stage of the study and collects and owns data, in some cases without engaging community partners [44]. To address this, WERA proposed the Community Owned and Managed Research Model [44], which consists of nine workgroups that collaborate to ensure equitable distribution of funds and control of data, emphasizing the credibility of community-based organizations to develop and manage research projects. WERA’s model encourages corrective actions to address and remove disparities for revitalization, sustainability, equity, and justice. It is another type of model to consider when conceptualizing future funding opportunities for community-engaged environmental health research.

## Conclusion

Workshop speakers explained the complex set of factors that lead to environmental injustices in communities, including environmental, spatial, ethical, political, cultural, social, and scientific issues. Speakers also elaborated on the contributing role of structural racism in environmental health disparities. Through conversations with community leaders, workshop participants learned applied research that addresses environmental injustices and health disparities must reflect the experiences of impacted communities, tackle the roots of environmental racism, and promote anti-racist practices. Outcomes from this research, including policy changes and other health protective recommendations, should not be instituted by academics, but by the organizations they serve — emphasizing the need for bidirectional communication and partnerships that involve people as equal partners.

This commentary not only illustrates the environmental justice issues faced by workshop speakers and the communities they serve, but also provides a model for others who hope to create effective partnerships between community organizations and academic researchers. Funded by NIEHS, the collaborative research projects described in this commentary are successful examples of how research that follows anti-racist principles and prioritizes affected communities can have tangible benefits and promote environmental health equity for all.

Although partnerships between communities and academics hold great promise for advancing environmental justice, there are still profound obstacles and difficulties, as stated by workshop speakers. Lessons learned from this workshop include that NIEHS and other institutions that fund research should redefine and rethink funding mechanisms, so they support activities of community-based organizations and invite communities into decision-making. We also learned the importance of communicating scientific research back to affected communities and to decision makers in a timely manner, of training the future workforce of anti-racist environmental health scientists, and of incorporating new technologies and data science to create solutions that cross the boundaries of scientific disciplines. The knowledge gained from this workshop provides a roadmap not only for NIEHS, but for other institutions across the world, to create new research programs that promote health equity, promote anti-racist principles within existing programs, and address environmental injustice at the root.
